# The use of complete-case and multiple imputation-based analyses in molecular epidemiology studies that assess interaction effects

**DOI:** 10.1186/1742-5573-8-5

**Published:** 2011-10-06

**Authors:** Manisha Desai, Denise A Esserman, Marilie D Gammon, Mary B Terry

**Affiliations:** 1 Department of Medicine, Division of General Medical Disciplines, Stanford University, Palo Alto, CA, 94304, USA; 2 Department of Medicine, Division of General Medicine and Epidemiology, and Department of Biostatistics, University of North Carolina School of Medicine, Chapel Hill, NC, 27599, USA; 3 Department of Epidemiology, University of North Carolina, Chapel Hill, NC, 27599, USA; 4 Department of Epidemiology and Herbert Irving Comprehensive Cancer Center, Columbia University, Mailman School of Public Health, NY, NY 10032, USA

## Abstract

**Background:**

In molecular epidemiology studies biospecimen data are collected, often with the purpose of evaluating the synergistic role between a biomarker and another feature on an outcome. Typically, biomarker data are collected on only a proportion of subjects eligible for study, leading to a missing data problem. Missing data methods, however, are not customarily incorporated into analyses. Instead, complete-case (CC) analyses are performed, which can result in biased and inefficient estimates.

**Methods:**

Through simulations, we characterized the performance of CC methods when interaction effects are estimated. We also investigated whether standard multiple imputation (MI) could improve estimation over CC methods when the data are not missing at random (NMAR) and auxiliary information may or may not exist.

**Results:**

CC analyses were shown to result in considerable bias and efficiency loss. While MI reduced bias and increased efficiency over CC methods under specific conditions, it too resulted in biased estimates depending on the strength of the auxiliary data available and the nature of the missingness. In particular, CC performed better than MI when extreme values of the covariate were more likely to be missing, while MI outperformed CC when missingness of the covariate related to both the covariate and outcome. MI always improved performance when strong auxiliary data were available. In a real study, MI estimates of interaction effects were attenuated relative to those from a CC approach.

**Conclusions:**

Our findings suggest the importance of incorporating missing data methods into the analysis. If the data are MAR, standard MI is a reasonable method. Auxiliary variables may make this assumption more reasonable even if the data are NMAR. Under NMAR we emphasize caution when using standard MI and recommend it over CC only when strong auxiliary data are available. MI, with the missing data mechanism specified, is an alternative when the data are NMAR. In all cases, it is recommended to take advantage of MI's ability to account for the uncertainty of these assumptions.

## Introduction

Recent advances in technology to measure biomarkers have given rise to increasingly more studies in molecular epidemiology. Consequently, many epidemiology studies now collect data from biospecimens for the purpose of studying the role of biomarkers in disease. Often these investigations assess synergistic effects between the biomarker and another feature on an outcome. A recent assessment of molecular epidemiology studies revealed that 30% of such studies evaluate a gene-environment interaction [1]. Availability of biospecimens such as blood or tissue samples, however, is generally limited to a subset of the subjects in the study, posing a missing data problem. Despite this, appropriate missing data methods are not typically being employed. In a 1995 study, Greenland and Finkle [2] attributed the underuse of missing data methods in epidemiology studies to their inaccessibility and complexity. Although missing data methods are more readily available at present, a recent study by Klebanoff and Cole in 2008 [3] found that less than 2% of papers published in epidemiology journals make use of more accessible missing data methods like multiple imputation (MI). Instead, a complete-case (CC) analysis continues to be the most widely applied method [1-4]. More specifically, a CC analysis excludes subjects missing data on at least one variable considered in the analysis. Desai et al. recently assessed the handling of missing data specifically in molecular epidemiology studies and found that while the majority of studies had missing data (65%) and/or excluded subjects with missing data from study entry (45%), 88% of these utilized a CC analysis [4].

The reasons underlying why the biospecimen data are missing matter. These may relate to observed features in the data set and/or the unobserved values of the biomarkers themselves. The statistical validity of CC methods (i.e., providing unbiased estimates and confidence intervals that achieve nominal coverage), however, relies on an assumption that the data are missing completely at random (MCAR); i.e., that missingness is unrelated to observed or unobserved data yielding a study sample that is representative of the larger cohort [5,6]. See Rubin for a more complete discussion on statistical validity [6]. If missingness is related only to observed variables (e.g., age), the data are considered missing at random (MAR). If, however, the reason for missing data is related to the unobserved values (e.g., even after conditioning on age, those with higher values of the biomarker are more likely to be missing biomarker data), the data are not missing at random (NMAR). CC analyses conducted on data that are not MCAR can lead to biased and inefficient estimates.

The data are limited in what they can reveal about missingness. Violation of the MCAR assumption can easily be investigated through simple comparisons of features between those with and without missing data. Without making unverifiable assumptions, however, it is impossible to distinguish between NMAR and MAR patterns, since the nature of missingness cannot be examined for data that do not exist. Thus, one must rely on assumptions based on biological, clinical and epidemiological understandings.

Theoretically sound methods for analyzing data under the MAR or NMAR conditions have been developed. For the former, this includes likelihood-based methods and standard MI [5], where MI is particularly simple to implement and readily available. For the latter, analogous methods (likelihood-based and MI-based) are available. These, however, are not as easily accessible, and are more complex to implement; unlike under the MAR condition, under the NMAR condition, one must model the missing data distribution. Valid likelihood-based methods for NMAR data include EM approaches to obtaining maximum-likelihood estimates and similar estimation strategies that exploit auxiliary data (defined as additional data that can be used to improve model performance given the missingness) [7-10]. While software has been developed for some cases under NMAR conditions, it has not been incorporated into mainstream statistical packages. Thus, access to specialized software presents a barrier to using these methods. MI, with the missing data distribution specified (such as pattern mixture models), is another alternative when the data are NMAR [11].

In molecular epidemiology studies there is often good reason to suspect the data are NMAR. For example, suppose those who consume more vegetables are more willing to provide blood samples and as a result, folate levels are measured less frequently on those with low levels. Furthermore, because it is not straightforward to distinguish between NMAR and MAR conditions, analysts may incorrectly assume the data are MAR. Such studies may also make auxiliary data available. A useful auxiliary variable is one that either correlates with the variable for which the data are missing, or one that correlates with missingness, or both. An example of the former may be the number of vegetable servings consumed, if it correlates with folate levels. An example of an auxiliary variable that correlates with missingness may be disease severity, if those with more advanced disease are more motivated to provide biospecimens. Finally, many molecular epidemiology studies focus on interaction effects, such as gene-environment effects. The bias and efficiency loss that results specifically from estimating these effects using a CC approach has yet to be characterized.

The goals of this paper are to characterize the performance of CC analysis when interaction effects are being assessed, and to discuss MI methods as a possible practical solution. We specifically investigate the consequences of applying MI, which in its standard form relies on the MAR assumption, and assess the extent that auxiliary data can help in estimation performance when data from a covariate are NMAR and the interest lies in estimating an interaction effect, involving the covariate. We examine situations when the covariate and therefore the modifying variable of interest are missing data and evaluate the impact of the strength of the auxiliary information under three conditions of missingness: large values of the covariate are more likely to be missing; extreme values of the covariate are more likely to be missing; and missingness depends on both the covariate and the outcome. We compare MI to the commonly applied CC approach on simulated and real data from a molecular epidemiology study.

## Analysis

### Multiple Imputation (MI)

MI is a simulation-based method for handling missing data. There are three main steps involved in conducting an MI-based analysis. The first step consists of imputing plausible values for missing data from a specified distribution. To incorporate the uncertainty of the imputed values, this is done *m *times to create *m *complete data sets, where *m *typically varies between 3 and 10. The data are analyzed separately for each of the *m *data sets in step 2, with the estimates appropriately combined to yield one summary result in step 3. The theoretical underpinnings of the method are described in Little and Rubin [5].

There are several approaches to specifying an appropriate distribution from which to draw the missing values required in the imputation step. In general, the strategies fall into one of two classes: the joint modeling approach or the fully conditional specification approach [12]. The joint modeling approach relies on specifying a joint density for the data to derive the posterior predictive distribution of the missing values [11]. The fully conditional specification approach, on the other hand, bypasses this step and imputes data on a variable-by-variable basis based on a specified conditional density. For more details on the comparison of these approaches see Van Buuren [12]. These methods are available in easily accessible software. SAS, for example, utilizes MI based on the joint modeling approach via the PROC MI and PROC MIANALYZE procedures. We provide example code that uses the fully conditional approach implemented via the ICE and MICOMBINE procedures developed by Patrick Royston for use in STATA [13-15] in Appendix A. Other software implementing MI can be found in Horton and Kleinman's comprehensive review [16].

### MI for Interaction Effects

Estimating interaction effects with MI is slightly more complicated than estimating main effects [17]. This has to do with the assumptions under which the data are imputed. More specifically, MI methods that rely on parametric assumptions such as a multivariate normal distribution may produce reasonable results for the estimation of linear relationships, but not for higher-order relationships. There are several approaches to imputing interaction terms. The two main approaches are to 1) impute the variables involved in the interaction first and then generate the product term for inclusion in the analytic model or 2) generate the product term prior to imputation and then impute this term like one would any other variable. These methods and others are discussed in detail by von Hippel [18]. We show example STATA code in Appendix A that implements both methods.

### Design of Simulation Studies

We assessed the performance of CC and MI methods for estimating interaction effects in two sets of simulation studies. In the first set, we evaluated their performance for estimating an interaction effect between two predictors (X1, which in some cases is continuous and in others is dichotomous, and a dichotomous predictor X2) on a dichotomous outcome (Y). One of the predictors (X1), and therefore the interaction term, is NMAR for a proportion of the subjects. An auxiliary variable (Z), generated as a linear function of X1 and random noise, is also available. The second set of simulations was performed to enhance our understanding of the impact of the nature of missingness using a more realistic set of auxiliary variables. To that end, we made use of a real molecular epidemiology study taken from the Long Island Breast Cancer Study Project (LIBCSP)[19,20], a population-based case-control study of breast cancer, where, for our purposes, the interest was in an interaction effect between alcohol consumption and presence of a mutant allele for being a fast metabolizer of alcohol as determined by the *ADH3 *genotype on breast cancer risk. Our simulations involved one variable with missing data (either genotype, which is dichotomous or alcohol exposure, which is either dichotomous or ordinal), a covariate of interest (either genotype or exposure) and a dichotomous outcome (case-control status).

Table 1 describes the thirteen scenarios examined. In the first set of simulations (Scenarios A-I) we evaluated the impact of [1] the nature of the missingness, and [2] the relationship between X1 and the auxiliary variable, Z, on estimation performance. To evaluate the impact of the nature of missingness, three conditions were considered. Under condition 1, the log odds of the probability of missing X1 is a linear function of X1. Under condition 2, X1 is more likely to be missing extreme values, that is, the log odds of the probability of missing X1 is a quadratic function of X1. Finally, under condition 3 the log odds of missingness is a linear function of X1 given Y. If Y represented case-control status, for example, this would allow cases and controls to differ with respect to missingness. In our study, cases are more likely to be missing large values of X1, and controls are more likely to be missing small values of X1. To assess the effect of the strength of the relationship between X1 and the auxiliary variable on the results, nonexistent, moderate and strong relationships were considered. Moderate strength was defined as a correlation between X1 and Z of 0.57 for X1 continuous and an increase of 1 unit in Z for X1 = 1 versus 0 for X1 dichotomous. For X1 continuous, a strong auxiliary variable was one that had a correlation of 0.97 with X1. For X1 binary, a strong relationship was defined as a 3 or 4 unit increase in the auxiliary variable when X1 was 1 versus 0. Each scenario is based on 1000 iterations with a sample size of 1000.

**Table 1:** Description of Scenarios Used in Two Sets of Simulation Studies.

Table	Set	Scenario	Median % Missing X1	Nature of Missing	Auxiliary Relationship	Type of Missing Variable
3a. Impact of Auxiliary Relationship Under Condition 1	1	A	22%	**Condition 1**^a^	**None^3^**	Binary

	1	B	22%	**Condition 1**^a^	**Moderate^4^**	Binary

	1	C	22%	**Condition 1**^a^	**Strong^5^**	Binary

3b. Impact of Auxiliary Relationship Under Condition 2	1	D	20%	**Condition 2**^b^	**None^3^**	Continuous

	1	E	20%	**Condition 2**^b^	**Moderate^6^**	Continuous

	1	F	20%	**Condition 2**^b^	**Strong^2^**	Continuous

3c. Impact of Auxiliary Relationship Under Condition 3	1	G	20%	**Condition 3^c ^**	**None^3^**	Continuous

	1	H	20%	**Condition 3^c^**	**Moderate^6^**	Continuous

	1	I	20%	**Condition 3^c^**	**Strong^2^**	Continuous

4a. Impact of Conditions Under Set of Auxiliary Variables with Varying Strength When Missing Genotype Data	2	J	21%	**Condition 1^d^**	**Realistic^7^**	Binary

	2	K	24%	**Condition 3^e^**	**Realistic^7^**	Binary

4b. Impact of Conditions Under Set of Auxiliary Variables with Varying Strength When Missing Exposure Data	2	L	21%	**Condition 1^d^**	**Realistic^7^**	Binary

	2	M	21%	**Condition 2^f^**	**Realistic^8^**	Ordinal

^a^Condition 1: X1 is 12.2 times more likely to be missing if X1 = 1

^b^Condition 2: Extreme values of X1 are more likely to be missing (probability of missing is a quadratic function of X1 or the log odds of missing X1 = *γ*_0_+*γ*_1 _X1 + *γ*_2 _X1^2^, where *γ*_1 _= -1 and *γ*_2 _= 2.)

^c^Condition 3: A 1-unit increase in X1 corresponds to a 7.4 times decrease in the probability of missing for controls, but a 7.4 times increase for cases

^d^Condition 1: Those with fast metabolizing genotype are 12.2 times more likely to be missing data on genotype. Missingness is also related to other observed covariates (mammogram, education, race, breastfeeding oral contraceptive use, hormone therapy use, and smoking status) as informed by the real data set for all subjects.

^e^Condition 3: Exposed controls with fast genotype are 7.4 times more likely to be missing genotype than those without, while exposed cases with fast genotype are 7.4 times less likely to be missing genotype. Unexposed subjects with fast genotype are 2.7 times more likely to be missing genotype. Missingness is also related to other observed covariates (mammogram, education, race, breastfeeding oral contraceptive use, hormone therapy use, and smoking status) as informed by the real data set for all subjects.

^f^Condition 2:Extreme values of exposure are more likely to be missing (probability of missing is a quadratic function of X1 or the log odds of missing X1 = *γ*_0_+*γ*_1 _X1 + *γ*_2 _X1^2^, where *γ*_1 _= -3 and *γ*_2 _= 1.) Missingness is also related to other observed covariates (mammogram, education, race, breastfeeding oral contraceptive use, hormone therapy use, and smoking status) as informed by the real data set for all subjects.

^1^Strong: Those with X1 = 1 have Z values (SD = 1), that are 3 units higher on average than those with X1 = 0

^2^Strong: Average correlation between X1 and Z is 0.97

^3^None: X1 and Z are independent variables

^4^Moderate: Those with X1 = 1 have Z values (SD = 1) that are 1 unit higher on average than those with X1 = 0

^5^Strong: Those with X1 = 1 have Z values (SD = 1) that are 4 units higher on average than those with X1 = 0

^6^Moderate: Average correlation between X1 and Z is 0.57

^7^Realistic: Auxiliary variables are informed by real data set and include all variables used to generate missing data mechanism of genotype (case-control status, exposure, mammogram, education, race, breastfeeding behavior, oral contraceptive use, hormone therapy use, and smoking status) as well as those that relate to genotype (exposure, race, breastfeeding behavior), a subset of the former.

^8^Realistic: Auxiliary variables are informed by real data set and include all variables used to generate missing data mechanism of exposure (case-control status, genotype, mammogram, education, race, breastfeeding behavior, oral contraceptive use, hormone therapy use, and smoking status) as well as those that relate to exposure (genotype, education, race, oral contraceptive use, hormone therapy use, and smoking status), a subset of the former.

Scenarios J-M comprise the second set of simulations and are also described in Table 1. These scenarios are informed by the real molecular epidemiology data set in two aspects that relate to the potential utility of the set of auxiliary variables: 1) the strength of the observed variables in describing missingness and 2) the strength of the observed variables in describing the variable with missing data. To determine these relationships a "fully observed" data set was created from the larger cohort. To create such a data set, subjects missing at least one of the variables of interest (case-control status, an indicator for having the genotype for fast metabolism, alcohol exposure, their interaction, and potential confounders of this relationship) were excluded, yielding a fully observed data set. The actual covariate values, and as a consequence, their inter-relationships were maintained in the simulations to enable comparison of methods under a more realistic and complicated set of auxiliary variables. New outcomes representing case-control status were generated as a function of genotype, alcohol exposure and their interaction.

Missingness was induced for genotype (Scenarios J-K) as well as for alcohol exposure (Scenarios L-M). This allowed assessment of methods under a realistic set of auxiliary variables under condition 2 (Scenario M: where extreme values of alcohol exposure are more likely to be missing), which only applies to variables with more than 2 levels and allowed comparison of methods under different sets of realistic auxiliary variables for condition 1. More specifically, there were fewer variables that correlated with genotype than with alcohol exposure with varying magnitudes of strength. We therefore compared methods across two sets of available auxiliary data for condition 1 (Scenarios J and L). Missingness was induced under three conditions analogous to the ones described above. In the first, the log odds of the probability of missing the variable (either genotype or exposure) is a linear function of the variable, as well as the following observed variables: the other covariate of interest (either exposure or genotype), ever having had a mammogram, education, race, having breast fed for at least 6 months, ever having been exposed to oral contraceptives, ever having been exposed to hormone therapy and smoking status. The second condition (Scenario M) specifies the log odds of the probability of missing alcohol exposure to be a non-linear function of exposure so that missingness is more likely for extreme exposures (none or high). Additionally, it is also a linear function of the observed variables mentioned above including genotype. Finally, the third condition specifies different missing data mechanisms conditional on genotype, exposure and case-control status (Scenario K). It specifies that exposed controls are 7.4 times more likely to be missing genotype if they have the fast metabolizing genotype, whereas exposed cases are 7.4 times less likely to be missing genotype if they have the fast metabolizing genotype and, unexposed subjects with the fast metabolizing genotype are 2.7 times more likely to be missing data on genotype. The log odds of the probability of missing genotype is also linearly related to the other observed variables considered above. The relationship between missingness and the observed variables is informed from the fully observed data set. Each scenario is based on 1000 iterations with a sample size of 2058, the size of the fully observed data set.

Three different models are presented: [1] the full model, which is the model fit on the complete data; [2] the CC model; and [3] the MI-based model, where the missing values of X1 were imputed as a function of X2, Y and the auxiliary variable, Z for the first set of simulations and as a function of the other covariate of interest (either exposure or genotype), ever having had a mammogram, education, race, having breast fed for at least 6 months, ever having been exposed to oral contraceptives, ever having been exposed to hormone therapy and smoking status for the second set. To produce optimal results, we set *m *= 10 as opposed to the more typical *m *= 5, although we found negligible differences when comparing the two. For each scenario, the data were analyzed using a logistic regression model with Y (case-control status) as the outcome and X1 (genotype), X2 (alcohol exposure) and their interaction as predictors. Average point estimates, average model-based standard errors (SE), average biases, mean squared errors (MSE), mean squared errors relative to CC (RelMSE), and percentage coverage with 95% confidence intervals were calculated for X1 given X2 = 0, X1 given X2 = 1, and their interaction. The comparison of MI to CC using the RelMSE statistic is critical, as a comparison of MI to the method currently used in practice (CC) is more relevant than its comparison to an optimal method. For an ideal reference, however, the full model is presented.

Table 2 describes the observed relationships between the potential auxiliary variables and the variable with missing data in the real data set. Candidate auxiliary variables for genotype (Scenarios J and K) include three variables that are highly correlated with genotype in the fully observed data set: alcohol exposure, race, and having breastfed for at least 6 months. For every increase in level of average alcohol exposure, a subject is 10% less likely to have the fast-metabolizing gene. Similarly those who have breastfed are roughly 30% less likely to have the fast metabolizing gene than those who have not. On the other hand, African American subjects have a 4-fold increased risk of having the fast metabolizing gene relative to Caucasians. There are a larger number of auxiliary variables for alcohol exposure (defined as alcohol consumer versus non-consumer), relevant for Scenarios L and M. These variables include race, where African Americans are less likely than Caucasians to be consumers, and where a higher education, use of oral contraceptives, hormone therapy and smoking exposure are all associated with a higher probability of alcohol exposure. Having the fast metabolizing, gene, however, corresponds to a 30% decreased risk in alcohol exposure.

**Table 2:** Description of Relationship Between Variable with Missing Data (Genotype or Exposure) and Set of Auxiliary Variables.

Variable with Missing Data:	Genotype (Scenarios J and K)		Exposure (Scenario L-N)	
Auxiliary Variables	OR	P-value	OR	P-value
Level of average alcohol exposure	0.90	P = 0.06	NA	NA
Race		P < 0.001		P < 0.001
Reference (Caucasian)	1.00		1.00	
African American	4.14		0.43	
Other	3.60		0.24	
Breastfed for at least 6 months		P < 0.001		
Reference (No)	1.00			
Yes	0.68			
Education				P < 0.001
Reference (less than high school)			1.00	
Completed high school and some college			1.52	
College graduate or more			1.68	
Use of oral contraceptives				P = 0.005
Reference (No)			1.00	
Yes			1.32	
Use of hormone therapy				P = 0.018
Reference (No)			1.00	
Yes			1.29	
Smoking Status				P < 0.001
Reference (Never smoked)			1.00	
Past smoker			1.63	
Current smoker			1.67	
Genotype	NA	NA		P = 0.001
Reference (No mutation)			1.00	
Has mutation			0.73	

### Example Data Set

As an illustration of these methods, we compared a previously published CC analysis of the gene-environment interaction of interest to one based on MI methods using data from the LIBCSP [19]. This particular analysis was undertaken to address a possible interaction effect between alcohol consumption and *ADH3 *genotype on breast cancer risk. Details of the overall study design are provided in prior publications [19,20]. In-person interviews were completed for 1,508 cases (82.1% of eligible cases) and 1,556 controls (62.8% of eligible controls). Seventy-three percent of both cases and controls who completed an interview donated a blood sample. As the CC approach adjusted for potential confounders, it further excluded those who were missing at least one variable and resulted in a data set of 1,008 cases and 1,055 controls. As previously published [19], subjects were more likely to donate blood if they were white, non-smokers, ever consumed alcohol, ever used hormone replacement therapy, breast-fed for six months or more, or ever had a mammogram.

### Results

The impact of the strength of the auxiliary variable for the three conditions generated under the first set of simulations, where approximately 20% of the data are missing, is shown in Table 3. For each condition, the first set of simulations examines three scenarios corresponding to non-informative, moderate, and strong auxiliary variables. These studies are supplemented by the second set of simulations shown in Table 4, which compares the methods under a more realistic and complicated set of auxiliary variables. Like the first set, roughly 20% of the data are missing. For completeness, estimates are presented for X1 given each level of X2 (denoted going forward as X1|X2) and their interaction. Performance, however, is based solely on estimation of X1|X2 = 0 and the interaction as their sum yields the estimate of X1|X2 = 1.

**Table 3:** Impact of Auxiliary Relationship Under Conditions 1, 2, and 3.

a.condition 1								
**Scenario**	**Variable**	**Method**	**Mean β**	**Mean SE**	**Mean Bias**	**MSE**	**RelMSE**	**Coverage**

A: No Auxiliary								

	X1 (X2 = 0) (True effect = 1.0)	Full	0.998	0.211	-0.002	0.043	0.403	95.2 (93.9,96.5)

		CC	0.999	0.323	-0.001	0.106	1.000	95.3 (94.0,96.6)

		MI	1.106	0.321	0.106	0.101	0.951	95.4 (94.1,96.7)

	X1 (X2 = 1) (True effect = 2.5)	Full	2.542	0.378	0.042	0.161	0.031	95.2 (94.0,96.6)

		CC	2.890	11.419	0.390	5.271	1.000	95.7 (94.4,97.0)

		MI	2.330	0.615	-0.170	0.317	0.060	92.8 (91.2,94.4)

	Interaction (True effect = 1.5)	Full	1.544	0.434	0.044	0.195	0.037	96.0 (94.8,97.2)

		CC	1.891	11.500	0.391	5.321	1.000	96.4 (95.2,97.6)

		MI	1.223	0.690	-0.277	0.382	0.072	94.6 (93.2,96.0)

B: Moderate Auxiliary								

	X1 (X2 = 0) (True effect = 1.0)	Full	0.986	0.211	-0.014	0.043	0.410	96.1 (94.9,97.3)

		CC	0.984	0.323	-0.016	0.105	1.000	95.7 (94.4,97.0)

		MI	1.157	0.312	0.157	0.110	1.039	94.9 (93.5,96.3)

	X1 (X2 = 1) (True effect = 2.5)	Full	2.53	0.376	0.03	0.149	0.066	95.9 (94.7,97.1)

		CC	2.698	4.867	0.198	2.28	1.000	96.1 (94.9,97.3)

		MI	2.438	0.600	-0.062	0.251	0.110	96.3 (95.1,97.5)

	Interaction (True effect = 1.5)	Full	1.544	0.432	0.044	0.195	0.080	95.7 (94.4,97.0)

		CC	1.714	4.949	0.214	2.437	1.000	96.8 (95.7,97.9)

		MI	1.281	0.671	-0.219	0.317	0.130	96.7 (95.6,97.8)

C: Strong Auxiliary								

	X1 (X2 = 0) (True effect = 1.0)	Full	0.997	0.211	-0.003	0.046	0.403	95.4 (94.1,96.7)

		CC	0.999	0.324	-0.001	0.113	1.000	94.4 (93.0,95.8)

		MI	1.027	0.223	0.027	0.051	0.447	95.3 (94.0,96.6)

	X1 (X2 = 1) True effect = 2.5)	Full	2.546	0.378	0.046	0.158	0.027	96 (94.8,97.2)

		CC	2.968	12.65	0.468	5.941	1.000	96.9 (95.8,98.0)

		MI	2.555	0.407	0.055	0.176	0.030	96.5 (95.0,97.4)

	Interaction (True effect = 1.5)	Full	1.549	0.434	0.049	0.200	0.034	96.1 (94.9,97.3)

		CC	1.969	12.730	0.469	5.971	1.000	96.6 (95.5,97.7)

		MI	1.528	0.462	0.028	0.205	0.034	96.2 (95.0,97.4)

b. Condition 2								

Scenario	Variable	Method	Mean β	Mean SE	Mean Bias	MSE	RelMSE	Coverage

D: No Auxiliary								

	X1 (X2 = 0) (True effect = 1.0)	Full	1.008	0.108	0.008	0.011	0.496	95.6 (94.3,96.9)

		CC	1.013	0.146	0.013	0.023	1.000	93.8 (92.3,95.3)

		MI	1.090	0.145	0.090	0.029	1.254	92.0 (90.3,93.7)

	X1 (X2 = 1) (True effect = 2.5)	Full	2.551	0.280	0.051	0.083	0.869	95.6 (94.3,96.9)

		CC	2.550	0.299	0.050	0.096	1.000	95.4 (94.1,96.7)

		MI	2.357	0.293	-0.143	0.085	0.888	93.1 (91.5,94.7)

	Interaction (True effect = 1.5)	Full	1.543	0.300	0.043	0.093	0.804	94.9 (93.5,96.3)

		CC	1.537	0.333	0.037	0.116	1.000	95.5 (94.2,96.8)

		MI	1.267	0.325	-0.233	0.126	1.084	90.2 (88.4,92.0)

E: Moderate Auxiliary								

	X1 (X2 = 0) (True effect = 1.0)	Full	1.011	0.108	0.010	0.012	0.533	95.4 (94.1,96.7)

		CC	1.009	0.146	0.009	0.023	1.000	94.5 (93.1,95.9)

		MI	1.163	0.142	0.163	0.046	2.039	81.4 (79.0,83.8)

	X1 (X2 = 1) (True effect = 2.5)	Full	2.558	0.280	0.058	0.090	0.911	94.2 (92.8,95.6)

		CC	2.555	0.298	0.055	0.099	1.000	94.5 (93.5,96.3)

		MI	2.571	0.296	0.071	0.085	0.855	96.7 (95.6,97.8)

	Interaction (True effect = 1.5)	Full	1.547	0.300	0.047	0.103	0.845	94.9 (93.5,96.3)

		CC	1.545	0.333	0.045	0.122	1.000	94.7 (93.3,96.1)

		MI	1.408	0.328	-0.092	0.102	0.832	95.1 (93.8,96.4)

F: Strong Auxiliary								

	X1 (X2 = 0) (True effect = 1.0)	Full	1.002	0.108	0.002	0.012	0.518	94.5 (93.1,95.9)

		CC	1.004	0.146	0.004	0.024	1.000	94.2 (92.8,95.6)

		MI	1.053	0.114	0.053	0.016	0.693	93.0 (91.4,94.6)

	X1 (X2 = 1) (True effect = 2.5)	Full	2.530	0.278	0.030	0.079	0.860	95.2 (93.9,96.5)

		CC	2.530	0.297	0.030	0.092	1.000	95.0 (93.6,96.4)

		MI	2.573	0.280	0.073	0.084	0.908	95.7 (93.8,96.4)

	Interaction (True effect = 1.5)	Full	1.527	0.298	0.027	0.092	0.788	95.0 (93.6,96.4)

		CC	1.526	0.331	0.026	0.117	1.000	94.9 (93.5,96.3)

		MI	1.520	0.303	0.020	0.092	0.791	95.1 (93.8,96.4)

c. Condition 3								

Scenario	Variable	Method	Mean β	Mean SE	Mean Bias	MSE	RelMSE	Coverage

G: No Auxiliary								

	X1 (X2 = 0) (True effect = 1.0)	Full	1.006	0.108	0.006	0.011	0.066	96.2 (95.0,97.4)

		CC	0.608	0.119	-0.392	0.168	1.000	10.2 (8.3,12.1)

		MI	0.680	0.119	-0.320	0.116	0.691	23.9 (21.3,26.5)

	X1 (X2 = 1) (True effect = 2.5)	Full	2.549	0.279	0.049	0.085	0.693	94.6 (93.2,96.0)

		CC	2.322	0.288	-0.178	0.122	1.000	85.3 (83.1,87.5)

		MI	1.974	0.274	-0.526	0.324	2.654	49.9 (46.8,53.0)

	Interaction (True effect = 1.5)	Full	1.543	0.299	0.043	0.097	0.640	95.1 (93.8,96.4)

		CC	1.714	0.312	0.214	0.152	1.000	92.3 (90.6,94.0)

		MI	1.294	0.297	-0.206	0.093	0.609	93.5 (92.0,95.0)

H: Moderate Auxiliary								

	X1 (X2 = 0) (True effect = 1.0)	Full	1.003	0.108	0.004	0.012	0.073	94.5 (93.1,95.9)

		CC	0.610	0.119	-0.390	0.167	1.000	11.9 (9.9,13.9)

		MI	0.793	0.116	-0.207	0.057	0.340	54.4 (51.3,57.5)

	X1 (X2 = 1) (True effect = 2.5)	Full	2.535	0.277	0.035	0.085	0.657	93.5 (92.0,95.0)

		CC	2.309	0.287	-0.191	0.129	1.000	85.1 (82.9,87.3)

		MI	2.222	0.279	-0.278	0.141	1.093	80.9 (78.5,83.3)

	Interaction (True effect = 1.5)	Full	1.531	0.298	0.031	0.096	0.658	94.3 (92.9,95.7)

		CC	1.699	0.310	0.198	0.146	1.000	91.4 (89.7,93.1)

		MI	1.428	0.301	-0.072	0.074	0.508	95.4 (94.1,96.7)

I: Strong Auxiliary								

	X1 (X2 = 0) (True effect = 1.0)	Full	0.999	0.108	-0.001	0.012	0.069	95.4 (94.1,96.7)

		CC	0.603	0.118	-0.397	0.172	1.000	11.1 (9.2,13.0)

		MI	0.986	0.109	-0.014	0.012	0.072	94.8 (93.4,96.2)

	X1 (X2 = 1) (True effect = 2.5)	Full	2.533	0.277	0.033	0.081	0.635	95.0 (93.6,96.4)

		CC	2.303	0.287	-0.197	0.128	1.000	84.2 (81.9,86.5)

		MI	2.533	0.278	0.033	0.081	0.628	95.2 (93.9,96.5)

	Interaction (True effect = 1.5)	Full	1.533	0.298	0.033	0.091	0.638	95.3 (94.0,96.6)

		CC	1.700	0.310	0.200	0.142	1.000	92.8 (91.2,94.4)

		MI	1.547	0.299	0.047	0.091	0.640	95.8 (94.6,97.0)

Results From Fitting Full, Complete-Case, and Multiple Imputation Models to 1000 Simulated Data Sets With a Sample Size of 1000 Where the Covariate of Interest and as a Result the Interaction Term Were Missing for Approximately 20% of Subjects.

**Table 4:** Impact of Nature of Missingness With Auxiliary Relationship Based on Data from LIBSCP.

a. Subjects Missing Data on Genotype Under Conditions 1 and 3								
**Scenario**	**Variable**	**Method**	**Mean β**	**Mean SE**	**Mean Bias**	**MSE**	**RelMSE**	**Coverage**

J: Condition 1	Effect of Fast Metabolizing Genotype For Unexposed (True effect = 1.0)	Full	1.001	0.155	0.001	0.025	0.738	95.1 (93.8,96.4)

		CC	0.999	0.182	-0.001	0.033	1.000	94.3 (92.9,95.7)

		MI	0.989	0.182	-0.011	0.032	0.954	95.0 (93.6,96.4)

	Effect of Fast Metabolizing Genotype For Exposed (True effect = 2.5)	Full	2.508	0.157	0.008	0.025	0.664	94.3 (92.9,95.7)

		CC	2.519	0.197	0.019	0.037	1.000	96.4 (95.2,97.6)

		MI	2.090	0.193	-0.410	0.190	5.144	42.4 (39.3,45.5)

	Interaction Effect (True effect = 1.5)	Full	1.507	0.220	0.007	0.049	0.684	94.7 (93.3,96.1)

		CC	1.520	0.268	0.020	0.072	1.000	95.5 (94.2,96.8)

		MI	1.101	0.265	-0.399	0.211	2.945	70.0 (67.2,72.8)

K: Condition 3								

	Effect of Fast Metabolizing Genotype For Unexposed (True effect = 1.0)	Full	1.003	0.155	0.003	0.022	0.666	96.1 (94.9,97.3)

		CC	1.000	0.185	0.0002	0.034	1.000	95.6 (94.3,96.9)

		MI	1.004	0.191	0.004	0.032	0.978	95.6 (94.3,96.9)

	Effect of Fast Metabolizing Genotype For Exposed (True effect = 2.5)	Full	2.509	0.157	0.010	0.024	0.024	95.9 (94.7,97.1)

		CC	3.463	0.233	0.963	0.983	1.000	0.2 (-0.1,0.5)

		MI	2.611	0.274	0.111	0.041	0.042	99.8 (99.5,100.1)

	Interaction Effect (True effect = 1.5)	Full	1.507	0.220	0.007	0.048	0.047	95.9 (94.7,97.1)

		CC	2.463	0.298	0.963	1.012	1.000	9.5 (7.7,11.3)

		MI	1.608	0.340	0.108	0.074	0.073	99.2 (98.6,99.8)

b. Subjects Missing Data on Alcohol Exposure Under Conditions 1 and 2								

Scenario	Variable	Method	Mean β	Mean SE	Mean Bias	MSE	RelMSE	Coverage

L: Condition 1	Effect of Fast Metabolizing Genotype For Unexposed (True effect = 1.0)	Full	1.016	0.155	0.016	0.025	0.962	94.7 (93.3,96.1)

		CC	1.015	0.158	0.015	0.026	1.000	95.4 (94.1,96.7)

		MI	1.308	0.150	0.308	0.115	4.502	45.6 (42.5,48.7)

	Effect of Fast Metabolizing Genotype For Exposed (True effect = 2.5)	Full	2.504	0.156	0.004	0.022	0.737	96.2 (95.0,97.4)

		CC	2.503	0.189	0.003	0.039	1.000	94.1 (92.6,95.6)

		MI	2.449	0.183	-0.051	0.026	0.835	96.9 (95.8,98.0)

	Interaction Effect (True effect = 1.5)	Full	1.488	0.220	-0.012	0.048	0.845	95.2 (93.9,96.5)

		CC	1.487	0.247	-0.013	0.056	1.000	96.4 (95.2,97.6)

		MI	1.141	0.247	-0.359	0.172	3.055	71.1 (68.3,73.9)

M: Condition 2	Effect of Alcohol Exposure for Those without Fast Genotype (True effect = 0.5)	Full	0.499	0.075	-0.001	0.006	0.622	95.5 (94.2,96.8)

		CC	0.501	0.095	0.001	0.009	1.000	94.6 (93.2,96.0)

		MI	0.560	0.095	0.060	0.012	1.348	91.4 (89.7,93.1)

	Effect of Alcohol Exposure for Those without Fast Genotype (True effect = 2.0)	Full	2.008	0.176	0.008	0.032	0.780	94.4 (93.0,95.8)

		CC	2.006	0.202	0.006	0.041	1.000	95.1 (93.8,96.4)

		MI	1.621	0.199	-0.379	0.169	4.183	50.5 (47.4,53.6)

	Interaction Effect (True effect = 1.5)	Full	1.509	0.192	0.009	0.037	0.743	95.2 (93.9,96.5)

		CC	1.505	0.223	0.005	0.050	1.000	95.6 (94.3,96.9)

		MI	1.061	0.217	-0.439	0.219	4.392	44.5 (41.4,47.6)

Results From Fitting Full, Complete-Case, and Multiple Imputation Models to 1000 Simulated Data Sets With a Sample Size of 2058 Where the Covariate of Interest and as a Result the Interaction Term Were Missing for Approximately 20% of Subjects.

#### Characterization of Performance of CC Analysis

In the first set of simulations (Table 3) under condition 1 (Scenarios A-C: where X1 is 12.2 times more likely to be missing for X1 = 1 than for X1 = 0), CC overestimated the interaction effect and gave average estimates of 1.7-2.0 when the true parameter was 1.5. In addition, CC analyses yielded large standard error estimates (ranging from 4.9 to 12.7), likely due to small cell counts when X1 is binary, missing for about 20% of subjects, and the proportion with X1 = 1 is small (0.2). In the second set of simulations, however, under an analogous condition 1 (Scenarios J and L: where genotype is 12.2 times more likely to be missing for those with the genotype of interest and missingness additionally relates to a set of observed variables), CC analysis performed better with respect to bias and efficiency. Although it still suffered from a loss in efficiency, the loss was much less dramatic than in the first set. Differences in how the data were generated between the sets contribute to the varying performance of CC under these similar conditions. In the first set of simulations the sample size (N = 1000) is half that of the second set (N = 2058). Additionally, the proportion of those with the genotype (or with X1 = 1) is 0.20 in the first set compared to 0.4 in the second set, where the latter proportion is determined by the real data set. Finally, in the second set, missingness also relates to additional observed variables. The performance of CC was comparable however between the two sets of simulations under conditions 2 and 3. Under condition 2, where extreme values of the variable are more likely to be missing, CC yielded approximately unbiased estimates on average for both sets of simulations but suffered a loss in efficiency; having the complete data set (full model) resulted in MSEs that were reduced by 50% for estimating X1|X2 = 0 and by 15-20% for the interaction effect. Under condition 3, CC performed the worst, underestimating the effect of X1|X2 = 0 in the first set of simulations (Scenarios G-I) yielding MSEs that were 14-15 times that of the full model and overstating the interaction effect in both sets of simulations, with MSEs that were approximately 1.5 and 20 times larger than that of the full model in the first and second set of simulations, respectively.

#### Performance of MI Relative to CC

Under all conditions shown in Table 3, when auxiliary information is strong (Scenarios C, F, and I), MI performed well and outperformed CC. Under condition 1 even when there is no auxiliary information, MI outperformed CC for both parameters, where the RelMSE statistic for the interaction effect showed a 93% improvement in estimation. Much of this improvement, however, is due to the inflated standard errors that CC provided due to small cell counts that occur in the presence of missingness when X1 is binary and the proportion with X1 = 1 is small. Under condition 1 in the second set (Scenarios J and L), however, CC was superior to MI. In both sets of simulations, MI underestimated the interaction effect and in the second, it provided poor coverage probabilities for the interaction effect, resulting in MSEs that were 3 and 2 times that of CC for Scenarios J and L, respectively. Under condition 2, MI needed a strong auxiliary variable to compete with CC. CC did not yield biased results, but suffered a loss in efficiency. With moderate to weak auxiliary information, MI underestimated the interaction effect and overestimated the effect of X1|X2 = 0. Overall it performed worse than CC and had an MSE that was 1.3 times greater than that of CC for X1|X2 = 0 in both sets of simulations and an MSE for the interaction that was 1.1 and 4.4 times greater in the first and second sets of simulations. The second set of simulations suggested that even under a realistic set of auxiliary variables, MI did not improve estimation over CC under this condition, where MI underestimated interaction effects. In both sets of simulations, MI improved performance over CC under condition 3. When there is no auxiliary data, MI and CC both underestimated X1|X2 = 0, but while CC overestimated the interaction effect, MI underestimated it. MI had a superior MSE statistic for both X1|X2 = 0 and the interaction. In the second set of simulations (Scenario K), these findings were supported. MI outperformed CC where CC overstated the interaction effect. MI performed well with good coverage probabilities and provided an MSE for the interaction effect that was 93% improved over that of CC.

#### Illustration on Real Data Set

The results from analysis of the LIBCSP evaluating an interaction effect between alcohol consumption and the *ADH3 *genotype on breast cancer risk are presented in Table 5. Adjusted odds ratios (ORs) from the previously published CC analysis, an MI-based analysis, and the percentage change in the beta coefficients or log-ORs for the interaction effect are shown. Both analyses involved fitting a logistic regression model adjusting for potential confounders (age at diagnosis; education; race; caloric intake; smoking status; and BMI). To impute values for genotype, these confounders as well as any variables related to missingness were used. These possible auxiliary variables included: having ever breastfed; having ever used hormone replacement therapy; having ever used oral contraceptives; ever having a mammogram; income level; and having benign breast disease. Terry et al. previously reported a two-fold association (OR = 2.3, 95% CI 1.3-4.0) for moderate alcohol consumption (15-30 g/day) for fast metabolizers using a CC approach. MI resulted in a 39% reduction in the coefficient (OR = 1.7, 95% 1.0-2.8) [19]. MI yielded parameter estimates that were smaller and closer to the null than those obtained by CC, where the percentage change in the beta coefficients ranged from 17% to > 100%, and the median percentage change was 31%.

**Table 5:** Results From Fitting Complete-Case and Multiple Imputation Models to Data from the Long Island Breast Cancer Study Project [19] Assessing the Effect of a Gene-Environment Interaction where m = 10

Genotype/Alcohol Status	OR^a^_CC_ N = 2,063 (95% CI)	OR^a^_MI_ N** = **3,064;m =10 (95% CI)	% Change in βCoefficient
Slow-Intermediate/Non-alcohol consumer	1.00	1.00	

Fast/Non-alcohol consumer	1.18 (0.88, 1.58)	1.14 (0.88, 1.48)	22.11%

Slow-Intermediate/< 15 grams	1.16 (0.89, 1.50)	1.11 (0.89, 1.39)	25.38%

Fast/< 15 grams	0.92 (0.69, 1.23)	0.95 (0.75, 1.20)	30.89%

Slow-Intermediate/15-30 grams	1.49 (0.99, 2.25)	1.27 (0.89, 1.82)	40.23%

Fast/15-30 grams	2.32 (1.35, 4.01)	1.68 (1.03, 2.75)	38.68%

Slow-Intermediate/30+ grams	0.72 (0.43, 1.21)	0.77 (0.49, 1.19)	17.40%

Fast/30+ grams	0.98 (0.52, 1.87)	0.86 (0.47, 1.56)	> 100%

CC = Complete Case; CI = Confidence Interval; MI = Multiple Imputation;OR = Odds Ratio

^a^Estimates are adjusted for age at diagnosis, education, race, caloric intake, smoking status and body mass index

## Conclusions

Studies of molecular epidemiology often involve collecting data on biomarkers. Issues with missing data arise when data are not fully observed for all subjects included in a study. The most common approach to analyzing these data is CC analysis [1-4], which has the advantage of computational ease but can result in estimates that are biased and inefficient. Using missing data methods in analyses, therefore, needs to become more customary. Standard MI is simple to implement and accessible but not recommended when the data are suspected to be NMAR. For example, while Taylor and colleagues promote using MI to reduce non-response bias in epidemiologic studies, they recommend doing so only when the MAR assumption is likely to hold [21]. In molecular epidemiology studies, however, one may suspect that the data are NMAR or one may incorrectly assume the data are MAR. In addition, many molecular epidemiology studies evaluate interaction effects, for which the bias and inefficiency of CC estimates have not been fully characterized. Our goal, therefore, was to characterize the performance under CC and to investigate standard MI, a method that is nearly as easy to implement and as accessible as CC, specifically in the context of assessing interaction effects when one of the predictors is NMAR.

### Characterization of Performance of CC Analysis

Biased and inefficient estimates from the CC approach were observed in our simulation studies, indicating a strong need for missing data methods. The extent to which they were observed, however, varied by the nature of missingness. When missingness is a function of the covariate only and not of the outcome (as in conditions 1 and 2) the performance of CC methods largely suffered in terms of efficiency loss. This loss in efficiency increased as the percentage missing increased from 20-40% (results shown in Table 6: Appendix B). Specifically, for condition 1, the MSE from the CC analysis was 12 times that of the full model when only 20% are missing and 125 times that of the full model when 40% are missing. For condition 2, the MSE of CC relative to the full model increased from 1.15 (when 20% are missing) to 1.78 (when 40% are missing). When missingness is a function of both the covariate and outcome (as in condition 3), the bias from CC was the most dramatic where it underestimated the effect of X1|X2 = 0 and overestimated the interaction effect.

#### Comparison of MI and CC Approaches in the Simulation Studies

Differences between MI and CC analyses varied by both the nature of the missingness and the strength of auxiliary information. Under all conditions, when auxiliary information is strong, MI outperformed CC. Interestingly, however, under certain conditions MI gave results that were more misleading than CC. Specifically, when large values of the covariate are more likely to be missing under a realistic set of auxiliary variables, MI was more biased than CC and underestimated interaction effects. Also, when extreme values are more likely to be missing and auxiliary information is not strong, MI was more misleading than CC. MI was superior to CC, however, when missingness relates to both the covariate and the outcome even under moderate and realistic sets of auxiliary variables, where CC overestimated interaction effects.

#### Application of MI and CC Approaches to LIBCSP

In the LIBCSP example, data were not MCAR as blood donation was related to a variety of observed factors [19]. We suspected that the data may be NMAR as having the genotype for fast metabolism was related to alcohol intake, and alcohol intake was associated with providing a blood sample. If after conditioning on alcohol intake, data on metabolism status were more likely to be missing for fast metabolizers (i.e., if other unmeasured features related to metabolism correlated with missingness) the data would be NMAR. While inference was similar between the two analytic approaches (the overall interaction effect was not statistically significant by either method) and consistent with previous findings [22,23], MI estimates were attenuated toward the null relative to CC. If missing genotype were related to observed features only, we would have more faith in the MI-based results as they rely on a more flexible assumption about the missingness than the CC method, which relies on an assumption shown to be violated in this case. Based on the findings of our simulation study, however, if genotype is also more likely to be missing for fast metabolizers (condition 1), we may be more likely to believe our CC results. If, however, missing genotype were to additionally relate to both genotype values and case-control status (condition 3), we may be more likely to believe the MI results. It makes sense in cases when CC and MI results are discrepant to present both analyses. The analysis that corresponds to the most plausible set of assumptions should serve as the primary analysis. Furthermore, a more comprehensive sensitivity analysis that presents results by varying assumptions about missingness can be performed using MI and is discussed in greater detail below.

#### MI, the MAR Assumption, and Its Relationship to Auxiliary Variables

The intuition behind why MI performed well when auxiliary data were strong has to do with the MAR assumption. Assuming the data are MAR is equivalent to assuming that the information needed to impute the missing values can be found in the observed data. This is a more reasonable assumption when the data include auxiliary information that is strongly related to the unobserved data. Thus, even if one were to suspect the data are NMAR, the presence of strong auxiliary information may allow one to proceed with methods that assume MAR.

Our simulation study is limited in that it does not provide a precise definition for the strength of the relationship necessary for one to assume MAR. In our simulations, for X1 continuous, a strong auxiliary variable was one that had a correlation of 0.97 with X1. For X1 binary, a strong relationship was defined as a 3 or 4 unit increase in the auxiliary variable when X1 was 1 versus 0. Although extreme and perhaps not likely outside of longitudinal studies, we felt it was important to study the extremes (no association and strong association) in addition to a moderate association (in our study, a correlation of 0.57 for X1 continuous and an increase of 1 unit in Z for X1 = 1 versus 0 for X1 dichotomous). To enhance our understanding, we supplemented the first set of simulations with another set based on the real molecular epidemiology data set that maintained the actual relationships between the variable with missing data and other observed data. Table 2 shows the relationship on the fully observed data between the variable missing data and observed variables. Not surprisingly, these relationships were altered in the observed data sets, with some variables not appearing to correlate as they did in the complete data, demonstrating the challenge of assessing good auxiliary variables from an observed data set. We therefore recommend coupling observations from the data with a priori knowledge when making assumptions about auxiliary relationships. Below we give specific guidelines to use in practice.

#### Practical Considerations

For detailed guidelines, we refer the reader to a recent study on the handling of missing data for molecular epidemiology studies, where Desai and colleagues provide greater details on the steps involved in a data analysis in the presence of missing data [4]. Below we emphasize some key points.

##### Making Plausible Assumptions

The first step when doing an analysis in the presence of missing data is to consider plausible assumptions about the missing data mechanism or reasons why the data may be missing. As the data themselves can only indicate whether the MCAR assumption should be ruled out, this will largely involve drawing on strong a priori knowledge. If there is no evidence that the MCAR assumption is violated (through a simple comparison of features between those with and without the missing variable(s)) and if based on clinical understandings, there is no reason to suspect the data are NMAR, MCAR may be assumed. As mentioned above, assuming the data are MAR is equivalent to assuming what needs to be learned about the missing data can be gleaned from the observed data. Thus, even if the data are NMAR, the presence of a strong auxiliary variable may make the MAR assumption reasonable. Similarly, even if MAR is more plausible than NMAR, one needs to consider which auxiliary variables allow this assumption to reasonably hold.

##### Choosing Appropriate Analytic Tools

A CC analysis should be performed in all cases. Additional tools, however, should be used and may serve as the primary analysis depending on the assumptions that are most likely. If the proportion missing is small (say less than 10%), a CC analysis should suffice. If the proportion missing is larger and there is evidence the data are not MCAR, one should incorporate a missing data method in addition to performing a CC analysis. If MAR is plausible or NMAR is suspected in the presence of strong auxiliary variables, standard MI is a reasonable choice. Otherwise, as we saw in the simulation study, standard MI can be misleading and under certain conditions, worse than CC methods. MI, with the missing data mechanism explicitly specified, such as pattern mixture models or selection models, is an appropriate choice in these situations and is discussed in greater detail in other sources [11]. Such an approach requires making explicit assumptions about the nature of missingness.

##### Making Use of Auxiliary Variables

As mentioned above, the choice of which auxiliary variables to include plays an important role in estimation performance of MI and the MAR assumption. In simulation studies described by Collins et al. [24], where this very issue was assessed for the MAR case, being more inclusive even when doubtful of the usefulness of some auxiliary variables resulted in increased efficiency and reduced bias.

##### Performing a Sensitivity Analysis

A nice feature of MI is its ability to incorporate the uncertainty of assumptions into the results, where the assumptions may involve the missing data mechanism (NMAR and MAR) as well as which auxiliary variables to include. One should perform a sensitivity analysis that involves presenting results using different subsets of auxiliary variables in the MI analysis, or in the case where MI is used after explicitly modeling the missing data mechanism, findings resulting from various assumptions of the missing data mechanism that should include multiple models under the NMAR mechanism. This will give a sense of the robustness of the results. The CC analysis should be included among these. If results across analyses differ, the investigator must decide which sets of assumptions are most plausible. Additionally, one can average over the resulting findings to provide one summary result that accounts for the uncertainty of the assumptions involving the missing data mechanism and/or choice of auxiliary variables.

##### Summary

In summary, molecular epidemiology studies face a particularly challenging missing data problem in that the majority of these studies will be missing data on the key variable of interest, the biomarker. While it may seem sensible to study only those with the measured biomarker, we argue the importance of including those who would be eligible for study despite the missing biomarker. At the very least, we urge comparison of features between those with and without missing data and strongly encourage the incorporation of missing data methods into the analysis when it is warranted. More specifically, if these comparisons indicate the data are not MCAR, and MAR seems plausible, we highly recommend use of standard MI. Even in cases where the data are MCAR, one can benefit in efficiency from MI. If it is likely that the data are NMAR and one can assume the strong presence of auxiliary information, standard MI may still be a reasonable estimation-enhancing tool. Otherwise, MI that models the missing data mechanism is a possibility. In all cases, a useful feature of MI is that it allows for incorporation of uncertainty of the missing data mechanism into the results.

## Competing interests

The authors declare that they have no competing interests.

## Authors' contributions

MBT conceived of the idea and helped to draft the manuscript. MD designed and carried out the simulations, interpreted the results, and helped to draft the manuscript. DE conducted the literature review and helped to draft the manuscript. MG provided insightful comments, suggestions, and edits, and led the parent study of the original molecular epidemiology study. All authors read and gave their final approval of the manuscript.

## APPENDIX A: STATA Code for Implementing MI

/* case is a binary indicator for case/control status, x1 and x2 are binary variables, where x1 is missing data on 20% of subjects and is NMAR and z is a continuous auxiliary variable */

/*Read in data set where data were generated under condition 1*/

insheet using "~/scen1.csv",

clear

/* Method 1 for Imputing Interaction Effects: Generate interaction term first and then impute */

/*Create Interaction term*/

gen theint = x1*x2

/*Use ICE to fit imputation model and create 10 imputed data sets*/

ice case x1 x2 theint z, saving(simimpute.dta) m (10) replace

/*Read in data set containing all 10 imputed data sets*/

use simimpute.dta, clear

/*Use MICOMBINE to fit the desired scientific model and combine results across 10 data sets*/

micombine logit case x1 x2 theint

/* Method 2 for Imputing Interaction Effects: Impute first then create interaction term as is done in passive imputation */

/*Create Interaction term*/

gen theint = x1*x2

/*Use ICE to fit imputation model and create 10 imputed data sets*/

/* Use passive option to implement Method 2 for imputing interaction term */

ice case x1 x2 theint z, saving (simimpute.dta) m (10) passive (theint:x1*x2) replace

/*Read in data set containing all 10 imputed data sets*/

use simimpute.dta, clear

/*Use MICOMBINE to fit the desired model and combine results across 10 data sets*/

micombine logit case x1 x2 theint

## APPENDIX B

**Table 6:** Impact of Percentage Missing Under Conditions 1, 2, and 3.

a. Condition 1^a^								
Scenario	Variable*	Method	Mean β	Mean SE	Mean Bias	MSE	RelMSE	Coverage

A1: 20% missing								

	X1 (X2 = 0) (True effect = 1.0)	Full	1.002	0.211	0.002	0.044	0.543	95.0 (93.6,96.4)
		CC	1.000	0.284	-0.000	0.081	1.000	95.8 (94.6,97.0)
		MI	1.062	0.230	0.062	0.052	0.644	95.9 (94.7,97.1)
	X1 (X2 = 1) (True effect = 2.5)	Full	2.549	0.378	0.049	0.166	0.068	95.4 (94.1,96.7)
		CC	2.713	4.822	0.213	2.436	1.000	97.0 (95.9,98.1)
		MI	2.543	0.426	0.043	0.178	0.072	96.8 (95.7,97.9)
	Interaction (True effect = 1.5)	Full	1.547	0.434	0.047	0.204	0.082	95.7 (94.4,97.0)
		CC	1.713	4.894	0.213	2.503	1.000	96.7 (95.6,97.8)
		MI	1.482	0.483	-0.018	0.205	0.082	96.1 (94.9,97.3)
A2: 30% missing								
	X1 (X2 = 0) (True effect = 1.0)	Full	1.003	0.211	0.003	0.044	0.358	94.6 (93.2,96.0)
		CC	0.992	0.345	-0.008	0.122	1.000	95.0 (93.6,96.4)
		MI	1.095	0.245	0.095	0.065	0.531	94.9 (93.5,96.3)
	X1 (X2 = 1) (True effect = 2.5)	Full	2.558	0.379	0.058	0.146	0.014	97.2 (96.2,98.2)
		CC	3.288	25.207	0.788	10.689	1.000	98.5 (97.7,99.3)
		MI	2.557	0.462	0.057	0.178	0.017	98.3 (97.5,99.1)
	Interaction (True effect = 1.5)	Full	1.555	0.435	0.055	0.195	0.018	96.1 (94.9,97.3)
		CC	2.295	25.290	0.795	10.904	0.018	96.1 (94.9,97.3)
		MI	1.462	0.515	-0.038	0.219	0.020	97.8 (96.9,98.7)
A3: 40% missing								
	X1 (X2 = 0) (True effect = 1.0)	Full	1.020	0.211	0.020	0.045	0.258	95.6 (94.3,96.8)
		CC	0.991	0.411	-0.009	0.175	1.000	95.5 (94.2,96.8)
		MI	1.137	0.263	0.137	0.083	0.474	94.1 (92.6,95.6)
	X1 (X2 = 1) (True effect = 2.5)	Full	2.540	0.377	0.040	0.140	0.006	96.6 (95.5,97.7)
		CC	4.121	64.794	1.621	23.920	1.000	96.5 (95.4,97.6)
		MI	2.536	0.489	0.036	0.177	0.007	97.5 (96.5,98.5)
	Interaction (True effect = 1.5)	Full	1.520	0.433	0.020	0.191	0.008	96.0 (94.8,97.2)
		CC	3.131	64.890	1.631	24.283	1.000	97.0 (95.9,98.1)
		MI	1.399	0.538	-0.101	0.220	0.009	97.2 (96.2,98.2)

b. Condition 2^b^								

Scenario	Variable	Method	Mean β	Mean SE	Mean Bias	MSE	RelMSE	Coverage

A4: 20% missing								
	X1 (X2 = 0) (True effect = 1.0)	Full	1.007	0.108	0.007	0.012	0.590	95.5 (94.2,96.8)
		CC	1.010	0.147	0.010	0.021	1.000	95.9 (94.7,97.1)
		MI	1.059	0.115	0.059	0.017	0.820	92.5 (90.9,94.1)
	X1 (X2 = 1) (True effect = 2.5)	Full	2.552	0.279	0.052	0.089	0.909	93.7 (92.2,95.2)
		CC	2.548	0.298	0.048	0.098	1.000	93.4 (91.9,94.9)
		MI	2.593	0.281	0.093	0.095	0.966	94.5 (93.1,95.9)
	Interaction (True effect = 1.5)	Full	1.544	0.300	0.044	0.102	0.872	93.6 (92.1,95.1)
		CC	1.538	0.332	0.038	0.117	1.000	94.0 (92.5,95.5)
		MI	1.534	0.304	0.034	0.101	0.863	94.0 (92.5,95.5)
A5: 30% missing								
	X1 (X2 = 0) (True effect = 1.0)	Full	1.003	0.108	0.003	0.012	0.437	94.5 (93.1,95.9)
		CC	1.008	0.168	0.008	0.027	1.000	94.6 (93.2,96.0)
		MI	1.079	0.118	0.079	0.020	0.741	92.4 (90.8,94.0)
	X1 (X2 = 1) (True effect = 2.5)	Full	2.532	0.277	0.032	0.078	0.798	95.7 (94.4,97.0)
		CC	2.529	0.317	0.029	0.098	1.000	96.2 (95.0,97.4)
		MI	2.612	0.284	0.112	0.091	0.928	95.6 (94.3,96.9)
	Interaction (True effect = 1.5)	Full	1.529	0.297	0.029	0.091	0.731	95.4 (94.1,96.7)
		CC	1.521	0.359	0.021	0.125	1.000	94.8 (93.4,96.2)
		MI	1.533	0.308	0.033	0.093	0.743	95.4 (94.1,96.7)
A6: 40% missing								
	X1 (X2 = 0) (True effect = 1.0)	Full	1.005	0.108	0.005	0.011	0.330	95.8 (94.6,97.0)
		CC	1.007	0.197	0.007	0.035	1.000	95.9 (94.7,97.1)
		MI	1.109	0.123	0.109	0.026	0.749	87.5 (85.5,89.5)
	X1 (X2 = 1) (True effect = 2.5)	Full	2.551	0.279	0.051	0.081	0.620	95.7 (94.4,97.0)
		CC	2.563	0.355	0.063	0.131	1.000	96.2 (95.0,97.4)
		MI	2.675	0.295	0.175	0.112	0.852	94.3 (92.9,95.7)
	Interaction (True effect = 1.5)	Full	1.547	0.300	0.047	0.094	0.563	95.1 (93.8,96.4)
		CC	1.557	0.406	0.057	0.168	1.000	95.3 (94.0,96.6)
		MI	1.565	0.319	0.065	0.098	0.588	96.9 (95.8,98.0)

c. Condition 3^c^								

Scenario	Variable	Method	Mean β	Mean SE	Mean Bias	MSE	RelMSE	Coverage

A7: 20% missing								
	X1 (X2 = 0) (True effect = 1.0)	Full	1.012	0.108	0.012	0.012	0.076	94.7 (93.3,96.1)
		CC	0.616	0.119	-0.384	0.162	1.000	11.9 (9.9,13.9)
		MI	0.999	0.109	-0.001	0.012	0.077	94.3 (92.9,95.7)
	X1 (X2 = 1) (True effect = 2.5)	Full	2.549	0.279	0.049	0.080	0.667	96.1 (94.9,97.3)
		CC	2.320	0.289	-0.180	0.120	1.000	87.1 (85.0,89.2)
		MI	2.551	0.280	0.051	0.080	0.666	96.2 (95.0,97.4)
	Interaction (True effect = 1.5)	Full	1.537	0.300	0.037	0.091	0.635	96.1 (94.9,97.3)
		CC	1.704	0.313	0.204	0.143	1.000	92.6 (91.0,94.2)
		MI	1.552	0.301	0.052	0.092	0.642	95.7 (94.4,97.0)
A8: 30% missing								
	X1 (X2 = 0) (True effect = 1.0)	Full	1.006	0.108	0.006	0.012	0.027	95.0 (93.6,96.4)
		CC	0.331	0.127	-0.669	0.466	1.000	0.3 (0,0.6)
		MI	0.983	0.110	-0.017	0.013	0.028	95.0 (93.6,96.4)
	X1 (X2 = 1) (True effect = 2.5)	Full	2.528	0.277	0.028	0.075	0.179	95.8 (94.6,97.0)
		CC	1.927	0.299	-0.573	0.419	1.000	48.6 (45.5,51.7)
		MI	2.492	0.277	-0.008	0.072	0.171	95.6 (94.3,96.9)
	Interaction (True effect = 1.5)	Full	1.522	0.297	0.022	0.084	0.743	96.0 (94.8,97.2)
		CC	1.596	0.325	0.096	0.113	1.000	95.7 (94.4,97.0)
		MI	1.509	0.298	0.009	0.081	0.713	96.1 (94.9,97.3)
A9: 40% missing								
	X1 (X2 = 0) (True effect = 1.0)	Full	1.007	0.108	0.007	0.011	0.012	95.8 (94.6,97.0)
		CC	0.027	0.139	-0.973	0.966	1.000	0.0 (0.0,0.0)
		MI	0.968	0.110	-0.032	0.013	0.013	94.1 (92.6,95.6)
	X1 (X2 = 1) (True effect = 2.5)	Full	2.537	0.277	0.037	0.079	0.092	95.6 (94.3,96.9)
		CC	1.627	0.312	-0.873	0.864	1.000	23.1 (20.5,25.7)
		MI	2.461	0.276	-0.039	0.073	0.085	95.4 (94.1,96.7)
	Interaction (True effect = 1.5)	Full	1.531	0.298	0.031	0.089	0.683	96.1 (94.9,97.3)
		CC	1.600	0.342	0.100	0.130	1.000	95.2 (93.9,96.5)
		MI	1.493	0.297	-0.007	0.081	0.627	96.9 (95.8,98.0)

Results From Fitting Full, Complete-Case, and Multiple Imputation Models to 1000 Simulated Data Sets With a Sample Size of 1000 Where the Covariate of Interest and as a Result the Interaction Term Were Missing for Some Subjects and the Auxiliary Information Was Strong.

^a^Condition 1: X1 is 7.4 times more likely to be missing if X1 = 1, where X1 is binary

^b^Condition 2: Extreme values of X1 are more likely to be missing (probability of missing is a quadratic function of X1 or the log odds of missing X1 = *γ *_0_+*γ *_1 _X1 + *γ *_2 _X1^2^, where *γ *_1 _= -1 and *γ *_2 _= 2), where X1 is continuous

^c^Condition 3: A 1-unit increase in X1 corresponds to a 7.4 times decrease in the probability of missing for controls, but a 7.4 times increase for cases, where X1 is continuous

## Authors' information

MD is the Director of the Quantitative Sciences Unit and Clinical Associate Professor in the Department of Medicine at Stanford University. DE is Research Assistant Professor in the Departments of Biostatistics and Medicine at the University of North Carolina at Chapel Hill. MG is Professor of Epidemiology in the School of Public Health at the University of North Carolina at Chapel Hill. MBT is Associate Professor of Epidemiology in the School of Public Health at Columbia University.
